# Dietary proteins, amino acids and insulin resistance: a mini review

**DOI:** 10.3389/fnut.2025.1671286

**Published:** 2025-10-20

**Authors:** Melynda S. Coker, Robert H. Coker

**Affiliations:** Montana Center for Work Physiology and Exercise Metabolism, University of Montana, Missoula, MT, United States

**Keywords:** metabolism, diet, signaling factors, nutrients, lipotoxicity and nutrition

## Abstract

The influence of protein intake on insulin resistance, has garnered an increasing amount of interest over the past few decades. Increased provisions of dietary protein during weight loss helps preserve skeletal muscle, which as the largest organ in the human body, is responsible for 80% of insulin-stimulated glucose disposal. The postprandial influence of essential amino acids (EAAs) either alone or as part of intact proteins are regulated through leucine-induced activation of mammalian target of rapamycin (mTOR) that serves to promote muscle protein synthesis and maintain skeletal muscle. High protein diets and/or EAA supplementation have also been demonstrated to improve satiety and augment mitochondrial function, which may have an indirect or direct influence on insulin sensitivity. On the other hand, chronic elevations in postabsorptive concentrations of branched chains amino acids (BCAAs) have been associated with chronic activation of the mTOR pathway, impairing insulin action. It appears that causal links between BCAAs and the pathogenesis of insulin resistance are reliant on chronic hyperinsulinemia and nutrient overload that foster chronic lipotoxicity. Conversely, postprandial elevations in EAAs leverage sensing as an anabolic mediator to facilitate muscle remodeling, augment satiety and improve metabolic regulation.

## Introduction

The influence of amino acids on insulin resistance is controversial. In this mini-review, we describe the differences between acute postprandial and chronic postabsorptive elevations in amino acids on metabolism. While environmental stress, physical stress and growth factors (ie., hormones, nutrients, and energy) influence signaling networks responsible for growth and proliferation, clinical outcomes also seem largely dependent on the energy status and metabolic health of the individual.

## Weight loss and improved insulin sensitivity: impact of high protein diet

Hypocaloric dietary interventions promote favorable changes in metabolic risk factors across a wide range of cohorts (1–5). Reducing energy intake promotes improvements in glucose metabolism in individuals with type 2 diabetes (6). While the impact of hypocaloric dietary interventions on the mitigation of insulin resistance is strong, dietary-induced weight loss can lead to muscle loss (7, 8). Based on conclusions from systematic reviews and meta-analyses, increased dietary protein during weight loss seems to preserve lean mass, accelerate the loss of fat mass, and improve cardiometabolic outcomes (9–13).

In a clinical trial that compared the influence of high-protein (i.e., 800 kcal, 45% protein, 35% carbohydrate, 20% fat) and high carbohydrate (i.e., 800 kcal, 20% protein, 60% carbohydrate, 20% fat) diets, the high protein diet promoted greater retention of fat free mass, improvements in insulin-stimulated glucose disposal (derived from euglycemic, hyperinsulinemic clamp method) and reduced 3-methylhistidine excretion (a marker of protein breakdown) compared to the high carbohydrate diet (14). Obese, insulin-resistant female participants who adhered to a hypocaloric high protein diet compared to a Mediterranean diet for only 3 weeks (15) demonstrated reductions in insulin resistance and fasting plasma insulin. Two-fold greater reductions in insulin resistance were described in participants with early-onset type 2 diabetes who followed a hypocaloric high protein diet (35% protein of caloric intake) compared to a hypocaloric standard protein diet (i.e., 18% protein of caloric intake) (9). Greater reductions in fat mass and insulin resistance have also been reported in overweight and obese women following a similar hypocaloric high protein (i.e., 35% protein of caloric intake) diet compared to a hypocaloric standard protein diet (i.e., 20% protein of caloric intake) (16).

When we consider that skeletal muscle is responsible for ~80% of insulin-stimulated glucose disposal and plays a key role in the etiology of insulin resistance (17, 18), modest elevations in dietary protein that preserve muscle mass during dietary-induced weight loss seem logical. The increased provision of dietary protein and EAAs in the context of a hypocaloric diet also fosters the preferential loss of adipose tissue that is directly proportional to the increased energetic demand linked to increased muscle protein synthesis (19). Based on the studies discussed above, mitigating the loss of skeletal muscle is a critical component of dietary interventions aimed at achieving reductions in body weight (20). Otherwise, caloric restriction-induced negative energy balance will result in 10–35% reductions in skeletal muscle, linked to reductions in functional capacity (7). Under these circumstances, muscle atrophy is especially problematic in older adults, leading to increased risk of falls, morbidity and mortality (21).

## Bed rest and insulin resistance: impact of high protein diet

Short-term bed rest (i.e., 10 days) promotes the onset of hepatic and peripheral insulin resistance in older, otherwise healthy adults as determined by multi-stage hyperinsulinemic, euglycemic clamp methodologies (22). Presentation of these metabolic abnormalities occurs in conjunction with the bed rest- induced loss of skeletal muscle and rapid decrements in functional parameters (19, 23). Even in young healthy adults, 3 days of bed rest resulted in a 45% decline in insulin-stimulated leg glucose uptake, coupled with a 43% reduction in whole-body protein and myofibrillar protein synthesis, and a 3% reduction in leg muscle volume (24). These detrimental alterations in metabolism and muscle remodeling reflect a reduction in energy demand and lack of mechanical stress to the skeletal muscle, resulting in perturbations in mitochondrial lipid metabolism (25). Glycogen and lipid intermediates accumulate under these conditions, which seems to foster elevations in fasting plasma insulin and insulin resistance (26). In the face of acute cessation of physical activity or bed rest, muscle atrophy, a decline in functional capability and the concomitant development of metabolic abnormalities are somewhat predictable, albeit unfortunate outcomes (22, 23, 27).

Resistance exercise has been demonstrated to mitigate the loss of skeletal muscle and the development of insulin resistance during bed rest (28). Physical activity is not always practical or well-tolerated in clinical settings (29), so dietary approaches to address the bed rest-induced loss of skeletal muscle and dysregulation of metabolism during bed rest have been investigated (30). Specialized approaches using branched-chain amino acids or individual EAAs have been effective in maintaining muscle protein synthesis and functional capability (31–33). Supplementation of ß -hydroxy-ß -methylbutyrate (HMB), a metabolite of leucine seems to promote mitochondrial function (i.e., OXPHOS complex II protein and total OXPHOS content) (34). Additional studies are needed to confirm these results and evaluate the protective influence of protein and/or EAA consumption against bed rest-induced muscle atrophy and the sequelae of insulin resistance (35).

## Amino acid supplementation, molecular mechanisms and metabolic health

Clinical evidence supports the role of EAA supplementation as a tool to improve metabolic health (36–39). EAA supplementation results in elevated mitochondrial biogenesis, reduced oxidative damage, enhanced muscle protein synthesis, physical capacity, reduced body weight, and improved immune function. A pharmaceutical therapy that promoted these types of systemic improvements would be heralded in the pursuit of health and longevity. It is therefore important to delineate the physiological mechanisms responsible for beneficial alterations in organ health that provide the above-stated EAA-related benefits.

To gain a better understanding of how these molecular mechanisms affect metabolic health, we should consider how the dietary consumption of EAAs influences metabolism in the muscle, liver, and adipose tissue. A sedentary lifestyle is commonly characterized by muscle atrophy, excessive accumulation of adipose tissue, and infiltration of fatty acids in the liver (40). The aging process itself further contributes to these metabolic abnormalities, partly due to the concomitant presentation of anabolic resistance, which makes maintaining a metabolically healthy balance between muscle and fat even more challenging. While increased physical activity is a powerful tool in the struggle against these abnormalities (41), only 20% of the adult population meets the guidelines recommended by the US Centers for Disease Control (42). EAAs, branched chain amino acids (BCAAs), or more specifically, leucine, have been consistently demonstrated to improve mitochondrial biogenesis in clinical and pre-clinical studies without any change in physical activity (36). The mechanistic underpinnings by which dietary consumption of amino acids mitigates pathogenic metabolic sequelae are then important.

**Figure 1:**
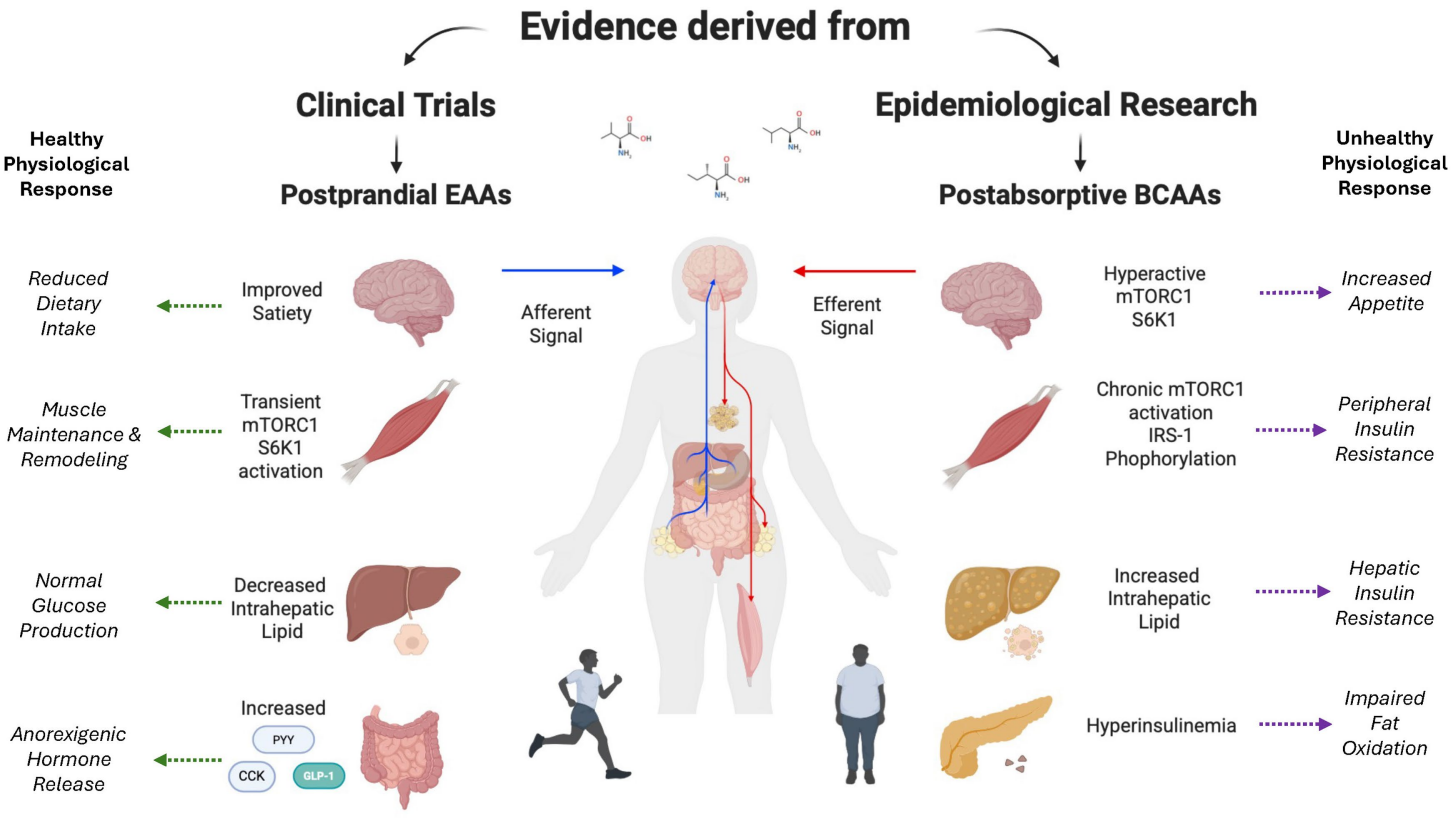
Dichotomous role of amino acids on insulin resistance. Impact of acute alterations in postprandial plasma EAA concentrations compared to chronic elevations in plasma BCAA concentrations on central and peripheral organ metabolism. Created with BioRender.com.

We know that amino acids, particularly leucine, transiently activate the mammalian target of rapamycin complex 1 (mTORC1), Yin-Yang 1 (YY1), and peroxisome proliferator-activated receptor-gamma coactivator-1-α (PGC-1α) (36) (Figure 1). Working cohesively with nuclear respiratory factors (NRF-1,2), mitochondrial transcription factor A (TFAM), and sirtuins (SIRT1/3), these signaling intermediates are responsible for the promotion of mitochondrial biogenesis (43, 44). On the other hand, reduced hepatic expression of PGC-1α has been closely linked to increased intrahepatic lipid in humans (Figure 1), whereas hepatic PGC-1α ablation in mice leads to compromised mitochondrial metabolism, which ultimately serves to rapidly induce hepatic steatosis (45). Perturbations in PGC-1α also contributes to the accumulation of ceramide in skeletal muscle, which has been implicated in the pathogenesis of peripheral insulin resistance (46). On the other hand, elevations in the expression of PGC-1α alleviate insulin resistance in skeletal muscle (47, 48).

The perturbed regulation of PGC-1α activation has been directly linked to several clinical problems. For example, disruptions in the PGC-1α/TFAM signaling pathway during pregnancy increase the risk of metabolic diseases in offspring (49). In patients suffering from diabetic kidney disease, mitochondrial dysfunction represents a hallmark of the clinical condition whereby PGC-1α activation is suppressed, potentially leading to the worsening of insulin resistance (50). Evidence from pre-clinical studies supports the role of PGC-1α and/or SIRT1 activation on the amelioration of lipotoxicity implicated in the pathogenesis of insulin resistance (51). PGC-1α also plays an important role in mitigating the detrimental impact of oxidative stress (52), which is directly connected to lipid abnormalities, atherosclerosis, hypertension, and increased risk of type 2 diabetes (53).

Physical activity, cold exposure, and/or caloric restriction are known to positively influence the regulatory influence of PGC-1α on mitochondrial biogenesis (54). Dietary-induced alterations in EAA availability, more specifically leucine, may also activate signaling pathways that augment mitochondrial biogenesis, potentially suppressing the development of insulin resistance even during periods of inactivity (55). Dietary leucine promotes browning of white adipose tissue and fatty acid oxidation in adipose tissue through the adenosine 5′-monophosphate-activated protein kinase (AMPK)-silent information regulator of transcription 1 (SIRT-1)-PGC-1α axis. In a somewhat similar fashion, dietary leucine activates PGC-1α through SIRT1-AMPK signals in skeletal muscle, increasing mitochondrial biogenesis, enhancing fatty acid and improving insulin sensitivity (55).

Therefore, the beneficial influence of EAAs on metabolic health may be derived from the augmentation of mitochondrial biogenesis that would not occur otherwise without increased physical activity (19, 23, 37, 38, 56, 57). While these changes appear to impact body composition and occur across organ systems in several clinical studies (58, 59), the EAA-induced activation of PGC-1α and the beneficial alterations in mitochondrial function may be the common denominator.

## Amino acids, satiety and metabolic health

The satiety cascade is regulated by a complex interplay between sensory factors, gastrointestinal influences via gastric distension and alterations in gut-derived peptides, and alterations in nutrient status/energy balance (60). The impact of these regulatory factors on food intake is consistent with the aminostatic theory whereby amino acids promote satiety (61) (Figure 1). In the context of dietary-induced elevations in amino acid concentrations, the regulation of nutrient intake may be directly influenced by the intrinsic need to maintain the effective remodeling of skeletal muscle. Dietary intervention studies linking amino acid concentrations to satiety support the theory (62, 63). On the other hand, pre-clinical studies suggest more comprehensive regulation, including the roles of glucoreceptors in the intestine and liver, playing a significant role (64). The influence of amino acids on satiety involves a complex network of central and peripheral nutrient sensing systems (65). While leucine-induced alterations in forebrain-hindbrain circuitry represent a central regulatory element that reduces nutrient consumption via negative feedback loops (66), divergent alterations in circulating anorexigenic hormones/orexigenic hormones elicited by dietary leucine highlight the importance of peripheral factors (67) (Figure 1).

Demonstrating the importance of peripheral regulation, high-protein diets have been demonstrated to elicit elevations in anorexigenic hormones while at the same time suppressing orexigenic hormones. Where dietary protein suppresses orexigenic hormones like ghrelin, anorexigenic hormones like GLP-1, cholecystokinin (CCK), and peptide YY are increased (Figure 1). For example, protein intake promotes GLP-1, which not only promotes satiety but has also been tied to enhancements in ß-cell function and glycemic status, which is logically consistent with the efficacy of GLP-1 as a therapeutic tool in the fight against metabolic disease (68). The amino acids phenylalanine and l-tryptophan are influential in promoting CCK secretion (69), which in turn serves to delay gastric emptying, promote satiety, and reduce dietary intake (70). In fact, the intravenous delivery of CCK reduces food intake, potentially influenced by the activation of CCK_1_ receptors that enhance satiety (71). While the specific amino acids responsible for the peptide YY response to protein have not been identified (72), acute dietary feeding of protein promotes the release of peptide YY that has a direct impact on improved satiation (73). Pre-clinical data also suggest that chronic elevations in dietary protein also increase plasma peptide YY and peptide YY expression (73).

Consistent with the importance of central regulation, leucine-induced activation of mTORC1 in the hypothalamus represents a crucial step in the central signaling cascade that influences satiety. Intracerebroventricular leucine administration reduces dietary intake, whereas administration of rapamycin ameliorates leucine-induced satiety (74). Downstream from mTOR, S6K1 activation has been demonstrated to reduce energy intake and mitigate metabolic perturbations, such as increased fat deposition and the presentation of insulin resistance, even during high-fat feeding in mice (75). High protein diets may also activate the noradrenergic-adrenergic neuronal pathway in the brainstem nucleus of the solitary tract and the meloanocortin neurons of the hypothalamic arcuate nucleus (75).

In addition to the impact of protein or amino acid intake on gut derived hormones that influence satiety, Skov et al., suggested that the inclusion of additional dietary protein also contributes to improved compliance to dietary interventions (76), ensuring negative energy balance required for weight loss that promotes improvements in insulin sensitivity (77). Given the multifaceted benefits of dietary protein on gut-derived hormones, it is not altogether surprising that evidence from dietary interventions support the role of high-protein diets in successful weight management, glucose homeostasis, and lipid metabolism (Figure 1).

## Controversial aspects of dietary protein and amino acids on the development of insulin resistance

A wide range of dietary interventions and/or EAA supplementation studies suggest definitive metabolic benefits (2, 9, 11–13, 15, 16, 19). On the other hand, elevations in fasting levels of BCAAs, sulfur amino acids, tyrosine, and phenylalanine have been closely linked to insulin resistance (78–80). These associations between fasting BCAAs and insulin resistance appear to strengthen over time (81), and BCAAs have even been suggested as potential biomarkers for predicting the risk of developing type 2 diabetes (82, 83) or for additional evidence of type 2 diabetes (84). Based on the results of a randomized controlled crossover trial, reductions in dietary consumption of BCAAs lowered meal-induced insulin secretion and postprandial insulin sensitivity as derived from the mixed meal tolerance test (85). However, direct measurements of hepatic and/or peripheral insulin sensitivity in these same studies were not affected by the reduction in dietary intake of BCAAs, suggesting the variations in splanchnic compared to peripheral glucoregulatory hormone concentrations between the mixed meal tolerance test and the clamp, respectively.

Insulin-mediated clearance of BCAA is impaired in obese individuals and seems to worsen in individuals with type 2 diabetes (86). Lower mitochondrial oxidation of BCAAs and reduced whole-body leucine oxidation rates were implicated in the chronic elevation of BCAAs (86). Hyperinsulinemia and nutrient overload may represent a physically inactive—hyperphagic phenotype. For example, chronic post-absorptive elevations in BCAAs have been linked to “hyperactive” mTOR and S6K1 signaling, impairing insulin action due to IRS-1 serine phosphorylation (80). 3-Hydroxyisobutyric acid, a catabolic intermediate of valine directly promotes fatty acid transport in skeleltal muscle, contributing to insulin resistance (87). Isoleucine also leads to increased muscle lipid deposition that has been linked to insulin resistance (88). Nevertheless, the causal link between BCAAs and the pathogenesis of insulin resistance is not clear. Inconclusive results from studies utilizing dietary supplementation of BCAAs, EAAs, and/or protein suggest the presence of multiple factors (i.e., source of intact protein, baseline physical activity, status of energy balance, etc) that play important roles, either negating the influence of fasting BCAAs or reducing their relevance to disease (89, 90) (Figure 1). Longitudinal clinical studies are needed to elucidate the reasons behind inconclusive results.

## Conclusion

The dichotomous role of protein, EAAs, and BCAAs in insulin resistance and metabolic health can be perplexing. A growing body of evidence supports the idea that high-protein diets and EAA supplementation support muscle retention, mitochondrial function, and insulin sensitivity, especially during periods of caloric restriction and/or physical inactivity. However, chronically elevated fasting plasma concentrations of BCAAs, under conditions of nutrient excess and hyperinsuliunemia, have been linked to overactivation of mTOR and S6K1 pathways, which may lead to systemic insulin resistance (Figure 1). These seemingly contradictory findings suggest that the relationships between dietary protein and a particular phenotype (ie., timing, context, culture, and metabolic status) may be responsible for the controversy surrounding dietary protein and metabolic health. This nuanced conundrum underscores the need for further investigation and discovery in amino acid metabolism. As we develop deeper insights into the timing of nutrient delivery, the state of energy balance, and cellular signaling, we should utilize amino acid-based strategies to offer targeted therapeutic potential in the prevention and management of insulin resistance and metabolic disease.
